# Post-Graduate Study

**Published:** 1910-09

**Authors:** J. M. Rattray


" *Scire est nescire, nisi id me*
*Scire alius sciret.*"

SEPTEMBER, 1910.

INDEX THROUGH

### POST-GRADUATE STUDY.

BY

J. M. RATTRAY, M.A., M.D., *President.*

The Inaugural Address at the Annual Meeting of the Bath and Bristol Branch of the British Medical Association, 25th May, 1910.

THE honour of addressing you at the oustet of another official year falls to me. I understand the rule in the election of president to be, out of three years one year for Bath, alternating with two consecutive years for Bristol. It is not very often that a country doctor is the recipient of the distinction, so I feel all the more deeply the honour which has been conferred upon me by the members of the branch and the Council of the Bath and Trowbridge divisions in my selection as president of the sixth largest branch of the British Medical Association. With the Association I can claim a long connection, having been a member for upwards of twenty-seven years, and having attended eighteen of the general meetings, besides many of the local ones. When I settled in Frome in 1882 my senior colleagues had not ceased to eulogise the splendid annual meeting of the Association held in Bath in 1878, under the presidency of Randall Wilbraham Falconer, M.D. One of the features of that meeting was the decision, taken on a vote, rejecting the admission of qualified medical women to the membership of the Association, a decision which was reversed at the Nottingham meeting in 1892.

The subject which I have selected for this occasion is Post-graduate Study . . . and here followed a retrospective personal allusion, ending with the statement that when the writer left, after a year's residentship in the Aberdeen Royal Infirmary in 1882, the principles of Lister had taken firm root, abdominal surgery was in full swing, deformities of the bones were being successfully treated by surgical operation, and Professor Ogston had discovered the micro-organisms of pus and certain surgical diseases.

With these preliminary remarks, I now proceed to lay before you a few thoughts which have long been present to my mind with regard to the subject of post-graduate study. We hear a good deal about the choice of a profession in a young lad's career, but not so much later on about a course of post-graduate study when he has qualified to enter the profession of his choice. Let me first of all say that if a man has received his degree or diploma, it is well that he should study, for a time at least, in the great school of general practice.

As "the three R.'s" made the embryo schoolboy, so the three great sections of practice—medicine, surgery and midwifery—make the embryo practitioner. At the outset, the practitioner's skill in these three subjects is not only unequally proportioned, but it is also of a very elementary description; therefore it is advisable that he should seek the opportunity of further hospital attendance, or of placing himself for a time in subordination to one or other of his more experienced colleagues. As adjuncts to his professional acquirements, I consider the newly-fledged practitioner should acquire a knowledge of drawing, shorthand, and cultivate the arithmetical faculty. Another excellent acquisition is some facility in discussion. The German student has the advantage over us in this: as a part of his medical examination he is trained to speak, for he must discuss a given subject for half an hour in every section of his examination.

The next, and most important acquisition for the young doctor is manual dexterity, although nowadays the necessity for rapid manipulation in surgery has gone. John Hunter, in his early days (as did also Sir William Fergusson, Bart., F.R.S.), tried his hand at cabinet-making, and prided himself on his manual dexterity. Lawson Tait, in like manner, told us in the address in surgery in 1890 that he served his time at the bench, and James Greig Smith gave the surgeon the name of "hand-worker." Although rapid operation work is no longer necessary, careful and exact handwork is more essential than ever, as many of our modern operative procedures are of a highly delicate and technical description. There are also many special departments of medicine and surgery which medical students are not required to attend for examination purposes, although during their professional career they find a knowledge of these to be essential. Again, much of the general practitioner's work is of a trifling nature, but it comprises duties demanding experience or watching the experience of others. Assistantship or pupilage is therefore a very valuable boon, as it enables the student to learn the management of patients, the art of prescribing, routine surgery work, to which may be added a knowledge of book-keeping, and nowadays particularly the art of driving and managing a motor-car.

While travelling abroad many years ago I met a Swiss doctor, who told me that for part of every year he was able to leave his practice and take a course of study at one or other of the great medical schools. Were we all able in this country to do likewise, what a boon it would confer on our professional life! Osler speaks of the "quinquennial brain dusting" at a hospital or post-graduate school, a process which is quite out of the power of the majority of practitioners in this country. For the country doctor's self-training, while he ploughs his lonely furrow, the means at his disposal are mainly reading, note-taking, the medical societies, and the methods of acquiring manual dexterity.

For reading, a good system of medicine and surgery is recommended, also a good cyclopædia for consultation, or the *Medical Annual*. In the larger towns the practitioner is better off, access to a good library being ready at hand.

When a medical man has regularly settled down in practice his post-graduate study resolves itself into two aspects: the one is that which has just been alluded to, viz. self-training; the other is attendance at a post-graduate college or other medical institution at stated periods. Looking first at the aspect of self-training, it is evident that in order to be a successful practitioner it is of the very highest importance that a considerable part of one's spare time be devoted to this object. Indeed, of the two forms of post-graduate study mentioned this is by far the more important, for the simple reason that a man who has kept his mental and practical faculties bright and polished can so much more readily assimilate and make good use of the information which he may pick up at a post-graduate college, and perhaps what is of still more importance, his interest and enthusiasm are maintained at a high level, after all the key to the easy and rapid acquisition of new facts and methods. A young doctor will find that at certain seasons of the year a great deal of spare time will be thrust upon him, and if he is wise he will at the outset of his career utilise it to keep himself abreast of the rapid changes and evolutions that occur in the progress of scientific knowledge.

As we are engaged in professional work that has not only a curative but preventive side, we have to deal not only with the necessities of the individual, but also with the means of arresting disease and the prevention of its spreading. Therefore the first and greatest essential is the matter of *diagnosis*. Success in practice, scientifically considered, depends chiefly on skill in diagnosis. Diagnosis involves a wide and careful study, in which the finer methods are developed, and the training of the special senses—the eye, the ear, and the sense of touch. And yet all these are impossible without an accompanying experience. It is when our special senses are constantly and minutely applied in our experience of disease that diagnosis tends to become more and more easy and perfect. The young practitioner is very often found to be capable in the diagnosis of what may be termed the classical diseases, but as a general rule he is only partially informed or quite uninformed on many subjects with which he would be called upon to deal in private practice ; but if he keeps a record of his experience noted down, and if he cultivates a good memory, he will be placed at a great advantage. In the practice of medicine memory counts for a good deal. In law a solicitor can look up his books in the presence of his client, or a barrister in consultation can look up the decisions of the courts. The client thinks all the more of his solicitor if he can read out the law to him from a book, whereas the doctor or consultant must appear as though his knowledge and opinions were the outcome of his own experience without turning to books or records, and the patient will be comforted when he learns that the doctor has had previous experience of his complaint. Hence the value of good note-taking in post-graduate study, and the practice of making comments on the scientific aspects and bearings of the notes that are begun. In this way, as Osler says, post-graduate study comes to be a habit of mind, and is acquired like other habits, viz. by the repetition of the practice of looking at things in an inquiring spirit. " Thus, a patient with pneumonia is found to have grass-green sputum. Have I ever seen it before ? Have I made a note of it ? If not, where can I get a good description of it, and what does it signify ? These are the sort of questions which lead to a good clinical education, which will stand the practitioner in good stead as the basis of intelligent judgment on many important cases."

After diagnosis we naturally think of *prognosis*, an element in post-graduate study which is to my mind the most difficult and the one least studied. Hippocrates gave it its value when he said : " It appears to me a most excellent thing for the physician to cultivate prognosis, for by foreseeing or foretelling in the presence of the sick the present, the past, and the future, and explaining the omissions which patients may have been guilty of, he will be the more readily believed to be acquainted with the circumstances of the sick, so that men will have confidence to entrust themselvses to such a physician, and he will manage the case best who has foreseen what is to happen from the present state of matters." This was the closing sentence of the address in medicine delivered by Sir Dyce Duckworth at the annual meeting at Carlisle, and I remember being much struck by a remark in that address where, quoting from an eminent French physician, the speaker said: "No one can frame a prognosis for a diabetic patient until he has been fifteen years in practice." This appeared to me at the time to be applicable to any other morbid condition; that prognosis became a matter of experience, and that we must look to the senior members of our profession only to forecast the issue of a case.

Here were quoted two cases, one to show the inaccuracy in prognosis that occasionally happens to the more experienced, and the other an exceptionally brilliant prediction by a surgeon of great celebrity.

Prognosis is the point which is most persistently put to the practitioner by the patient or his relatives. The first word is, "What do you think of the case?" and an answer is eagerly awaited, and when given its effect is decisive. Therefore it is a good rule that the practitioner, having once given his opinion, should stick to it, or confidence in him will be lost. Remembering the pitfalls of prognosis, great circumspection should be exercised by the practitioner in his choice of words where the case is doubtful. On the other hand, when the prognosis is clearly good, it ought to be made with confidence. Under all circumstances hopefulness should be the predominant note, and even in desperate cases the best should be seized upon and made a source of comfort.

A good illustration of the pitfalls in prognosis referred to is that afforded by tuberculosis of the lungs. If a patient on first examination were to show signs of cavities and other advanced signs of disease, the practitioner might be liable to state that the patient could not live more than a few months. Sometimes, however, inquiry into the history will elicit the fact that the patient had been suffering for years with cough and expectoration, and the case may be one of fibroid phthisis, with possibly twenty years of life in front of him. The late Sir Andrew Clark was fond of referring to this danger in the prognosis of phthisis, as he himself was a living example of it, having early in his life been condemned to die in six months, according to the opinion of an eminent physician of the time. Sir Dyce Duckworth's quotation, already referred to, struck me from the day I heard it, and even in one's small country experience one finds with diabetes that in early adult life it is a highly dangerous disease; that insuperable constipation in a middle-aged diabetic is a grave condition; and that suspicions of the onset of coma at any age may be taken as significant of approaching demise. Again, one was taught to consider the "typhoid state" as indicative of impending death, but one lives to see the dry, brown tongue, with, weak irregular pulse and delirium, recovered from, and the occurrence of Cheyne-Stokes respiration in cerebral hemorrhage need not necessarily portend a fatal issue. On the other hand, it is my experience that acute diseases of a serious nature occurring in a hemiplegic are generally fatal, and the sudden onset of general coldness in an aged person suffering from any disease affecting the vital organs may be taken as the precursor of imminent death. These personal aphorisms on prognosis, however, seem to tempt me from the subject of my paper, and to bring this department of medicine in line with post-graduate study would be to recommend the address on prognosis from which I have quoted, and generally to note the many circumstances that help one to gauge the constitutional and vital powers of the patient.

There is, as I have said, no greater test of a physician's skill in the eyes of the public than his ability to forecast the result of disease. There are few of us who cannot recount errors of prognosis made by ourselves or our friends. Many persons continue to live who were condemned to die, and many have died whose end was not expected. It is ever wise to exercise the greatest discretion, and I commend the dictum of the philosopher Bacon, who counselled "not to engage a man's self peremptorily in anything, though it seem not liable to accident, but ever to have a window to fly out at or a way to retire."

To prognosis succeeds the question of *treatment*, and for purposes of proficiency in private practice in this matter I ever advocate a forward policy. The subject of the address from this chair last year was the "*Vis Medicatrix Naturæ.*" Its influence I fully recognise; but we are here to cure or relieve disease or distress, and I for one am ever ready for profit to oblige the public in their craving for the multifarious medicaments and remedies whose names are now rendered so familiar by the lay authorities, so diligent and so enterprising in original research in this department of our calling.

For a long time there was something of the nature of a reaction against active medication, but in later years the fashion seems to have come in again. With regard to the question of drugs, we find at the present day a considerable modification of the older methods. In the old days nearly all drugs were compounded from pharmaceutical preparations and made up to the doctor's prescription. Some of them had the disadvantage, no doubt, of being rather nauseous, and this perhaps to some extent has accounted for the fashion which has been considerably overdone, of ordering proprietary preparations to the neglect of the regular prescription. While some of those preparations are very useful and conveniently made up, the neglect of the prescription is to be much deplored, and I would urge the careful consideration of this point. The present surfeit of proprietary articles is also fraught with considerable risk to the public, who get to know of them, and owing to their convenient form self-medication is encouraged to their and our detriment. Doctors in practice in the country still dispense, and although as a rule an assistant of some kind is available, many have personally to undergo the drudgery, and I think this has a tendency to lead to a routine system of prescribing, for one may have a desire to use up the stock in the surgery rather than base one's prescriptions on a rational therapeusis.

"We all have our therapeutic ruts," says Osler, "and we all know consultants from whom patients find it very difficult to escape without their favourite prescription, no matter what the malady may be." Personally, I limit myself to the *British Pharmacopœia*, which I tell my patients is sufficient for the wants of my practice; and if I can reflect the light of post-graduate study to improve the pharmacodynamical powers of any of my hearers, it would be to direct their attention to the section of general therapeutics in the excellent work of my friend Dr. Mitchell Bruce, keeping pace with which, edition by edition, has ever been my mainstay in trying to follow a rational therapeusis. There are few bookshelves of medical men who read that do not contain *A Manual of Medical Treatment, or Clinical Therapeutics*, by Burney Yeo, and as dieting is one of the first essentials in rational treatment, *Food in Health and Disease*, by the same author, will prove an excellent guide and form an element of the greatest advantage in post-graduate study.

I now come to the second method of study, or *post-graduate study proper*. This refers to the attendance from time to time of the practitioner at a post-graduate teaching centre; but before speaking strictly of this, I may just allude to the opportunities which the annual holiday gives of acquiring information and experience at the various health resorts, both home and foreign. It does not follow that those furthest afield are best to select; for instance, no station can be better equipped or more abreast of the times than is exemplified in this city of Bath. My friend Dr. George Oliver, of blood-pressure fame, states that the physician practising at a health resort may be regarded as a specialist in chronic ailments, and that chronic ailments are generally the outcome of some long-operating cause or causes, hereditary or individual, and the material which they furnish for his daily thought and work induces the physician to cultivate a trustful study of and reliance on physiology as the guiding star of his practice. One finds, however, that it is impossible to attain to such a high ideal as that, and the true scientific spirit is too often far beyond our reach in dealing with chronic diseases and affections of a monotonous nature.

The British Medical Association's annual general meeting has recently given facilities for visiting the health resorts of Harrogate and Buxton, and those who would care to increase their knowledge of the climatic health resorts abroad would find a very agreeable method through the *Voyage d'Etudes Médicales*, where there is open to medical men a yearly trip to the various departments of France and the health resorts in each.

*Post-graduate Schools.*—With regard to post-graduate study proper, or the attendance of the practitioner from time to time at one of the post-graduate centres, this has usually been the privilege of the wealthier and more leisured members of our profession. There was an old rule in the Scottish Universities that qualified men in practice for fifteen years could matriculate, attend for one year the hospital and University, and present themselves for the final examination for the M.D. degree. Here was a good opportunity for "brain-dusting." Generations ago the great masters of medicine spent years in foreign study, but the pace they set is a hard one to keep up.

Nowadays practitioners who wish to have the best the world affords can have it through the numerous opportunities at their disposal for acquainting themselves practically with the enormous advances in the science and art of medicine and surgery with their various branches. The type of school that combines the cream of the clinical wealth of the great schools of medicine for the provision of clinical teaching for qualified medical men would be represented by the Medical Graduates' College and Polyclinic. A member of this has the right to attend clinical instruction, operations, and necropsies at all the great hospitals included in the scheme. On the other hand, if the student desired to take up any particular speciality alone he could attach himself to one or more of the special hospitals, where he can see the class of cases he wishes to study every day, and have instruction on them from a variety of teachers. The West London Post-Graduate College devotes its clinical material solely to the instruction of qualified medical men. A feature of the course is instruction in the administration of anæsthetics, including spinal analgesia, on the operating days. The London School of Clinical Medicine, the North-East London Post-Graduate College, the Seaman's Hospital, and the great Medical Schools of Cambridge, Edinburgh, Glasgow, and Trinity College, Dublin, give ample facilities for post-graduate education.

Instruction at post-graduate colleges has direct action in the stimulation of a more thoughtful acquisition of medical knowledge. The ordinary five years' course can afford instruction of a text-book character, but post-graduate instruction leads to a more broadly thoughtful form of study. "My intercourse with those amongst them who had any conversational talents greatly stimulated my intellect," said De Quincey. In like manner thoughtful study is prompted by more advanced discussions, demonstrations and examinations of patients. These last are of special value in the acquisition of powers of diagnosis, and gradually one would be able to look back upon a considerable clinical experience instead of the mere mental recitations of text-book signs and symptoms required for a medical examination.

This difference in the character of the mental attitude of a student and a practitioner is very marked and very important. As a student one is obliged to cram much and to think little. The student does not correlate his pathology to his diagnosis and treatment. His mind is a store-house of facts. But as a practitioner he is brought to consider the why and the wherefore; his acquired facts have dovetailed with each other in the light of thought and experience; clinical demonstrations have a much more illuminating mental effect, and he acquires a general grasp of the science of medicine to which he was formerly quite a stranger. Hence the great value of a medical man endeavouring to set apart two or three weeks annually or biennially for this experience, which has the effect of constantly maintaining the scientific habit; it also adds a charm to practice which arouses enthusiasm and mental satisfaction.

*Bristol University.*—We have now got in the West of England that which has been so ardently desired for so many years—a well-equipped University with an abundant store of material. Time was when there was little else in England but the older Universities of Oxford and Cambridge, London and Durham; but modern universities have been established in several of the great provincial cities, and, thanks to the annual general meeting of the British Medical Association, we have been privileged to visit several of these modern schools of thought in Leeds, in

Manchester, in Birmingham and in Sheffield. A new University has, however, sprung up quite close to us, and will remain for all time. Its deed of Charter was approved last year, and the *Calendar* of the University sets forth its professions and its capabilities. For many years medical teaching was carried on at the Medical School which connected the Royal Infirmary and the General Hospital, but henceforth it will be carried on as the outcome of the establishment of the constituted Faculty of Medicine of the University.

We are now in a new era through the reformation of the medical curriculum. The President of the B.M.A., Sir William Whitla, in his address, partially entitled, "A Survey of the State of Medical Education," said: "It behoves us periodically to review our position as regards the teaching and the practical training of those desirous of joining our ranks," and "A medical curriculum which was planned forty years ago is entirely out of date." The latest reforms suggested are that anatomy should be taught from the standpoint of medical and surgical anatomy, and physiology with strict reference to practical medicine; the same direction towards their ultimate particular application is to be kept well in view in all the preliminary subjects. This programme accords with the most recent opinions of leading medical educationalists.

Post-graduate study at Bristol University is at present represented by a class held in the department of pathology, and attended chiefly by qualified men entering for the M.B. (Bristol). Intimation was given in the *British Medical Journal*[1] that a post-graduate course was about to be instituted in Bristol for qualified men who desired to read for higher degrees or diplomas, or who wished to improve their technique in surgery or surgical methods. But as our University gets under way the practitioners of the West of England will doubtless be well provided for in all the general and special departments of our science.

Gentlemen, my task is nearing completion. The remarks I have addressed to you were inspired by my predecessor's

[1] October 29th, 1904, p. 1192.

presidential address on the outlook of the medical student of the future, and the struggle which lies before him as knowledge extends and the necessity for closer study becomes greater. Extending this theme to the consideration of the lifelong studentship of the practitioner after he has completed his curriculum and obtained the imprimatur of his university or examining board, I have endeavoured to present some aspects of that lifetime of study and strenuous endeavour which he should set before himself. Hands, eyes, ears and head have to be trained, and experience to be set firmly on the "written tablets of the brain." "Write your own text-book of surgery," was the advice given in an address by Mr. Pearce Gould some years ago. This is surgical post-graduate study in a nutshell, and it might be extended to the other branches of medical science. The majority of us are unable to negotiate post-graduate courses, and with the calls made on our time and ability to take part in the general interests of the community where one is located, there is sometimes little opportunity even to fall back on books or magazine literature. Thus the danger of getting into a mental rut, or of coming under the domination of fixed ideas.

One has heard it said that we have often enough to do in endeavouring to keep pace with the original research of our patients. In country places the stimulating force is often the inquiring laity, who love to discuss matters medical which they pick up from all quarters, and nowadays in great part from newspapers. It is necessary to be abreast of all the latest medical fads, such as Christian science, faith-healing, etc., which a credulous public are only too willing to embrace. Those amongst us, on the other hand, who have the opportunity of attending a post-graduate hospital have the great advantage of seeing the application and results of the newer methods of treatment or operation. This is a very different matter to merely reading about them in journals. When the technique of these methods has been seen, and possibly to some extent acquired, the practitioner is placed in a much more advantageous position. He can then gauge in what cases he should adopt or recommend their application, and thus insight into the probabilities becomes clear. On the medical side I may refer to such methods as vaccine and serum-therapy, blood examinations, and the determination of the opsonic index ; radiology as a means of diagnosis and of cure, ionic medication, etc. It is also well for the practitioner to see the application, even should he not practise it himself, of the newer instruments, such as those for illuminating the internal cavities of the body, like the gastroscope and bronchoscope. Also how many useful and practical hints may not the practitioner obtain from seeing the latest developments in surgery and anæsthesia, such as the surgery of the viscera, which, as in the case of the heart, has attained such wonderful possibilities.

But, gentlemen, post-graduate study is not altogether confined to science. At the present time, more than at any other period in our profession, the medical man requires to be conversant with the economic problems of the hour. These have become very pressing, and affect medical practice in a vital manner. So important and essential has the study of these problems become to the medical man, that the British Medical Association has instituted for the first time this year a section entitled "Medical Sociology" at the annual meeting in London next July, when problems of high national importance will be discussed, such as the State insurance of sickness and invalidity, contract practice, the Poor Law medical service (which at present is causing such a commotion both inside and outside our ranks), hospital abuse and its prevention. It is highly desirable, therefore, to have these questions discussed at our local meetings, and some idea as to our position with reference to them defined.

It is evident, indeed, that the practitioner of the present day requires to be an all-round man, up to date both scientifically and socially. He has, therefore, to determine in the time at his disposal what is essential and what is non-essential. "Some books," says Bacon, "are to be tasted, others to be swallowed, and some few to be chewed and digested." It is unwise to admit too much, but on the other hand it is well to concentrate one's thoughts on the matters of everyday life, and to avoid theorising on what will probably never come under one's experience.

> "Not to know at large of things remote
> From use, obscure and subtle, but to know
> That which before us lies in daily life,
> Is the prime wisdom."—Milton, *Paradise Lost.*

Lastly, let it be said that he will labour most successfully who is ready in due course and at the right time to relax his mental strings, and to promote his physical and mental welfare by healthy and manly recreations. Severe and sustained effort can be maintained for only a limited period, and without islets of repose in the midst of the seas of effort, the hardiest worker will find that his best efforts are imperfect and unsatisfying, and his work will lack the spring and freshness of a healthful energy.

> "The heart of man, walk it which way it will,
> Sequestered or frequented, smooth or rough,
> Down the steep valley among tinkling flocks,
> Or 'mid the clang of trumpets and the march
> Of clattering ordnance, still must have its halt,
> Its hour of truce, its instant of repose,
> Its run of rest."—Sir Henry Taylor's *Philip van Artvelde.*

As medical practitioners, we not only require the annual holiday, however short, but we require day by day a little time devoted to some relaxation or hobby, the pursuit of which tempers and balances the professional bent. It is where the spirit, the soul and the body are exercised and trained in unison that the best results are obtained. Where one or other is over-stimulated on the one hand, or neglected on the other, we have to suffer the consequences. This, however, is a departure into the domain of psychology, and seeing that the sands of the time allotted to me have already run out, I conclude this address with a quotation which I think is peculiarly applicable—

> "Lo, thirty centuries of Literature
> Have curved your spines, and overborne your brains!
> Off with it, all of it! Stand up, behold
> The earth, life, death, and day and night.
> Think not the things that have been said of these,
> But watch them and be excellent, for men
> Are what they contemplate."
> John Davidson, *Self-Instruction by Experience.*

